# Phenolic Compounds and Antioxidant and Anti-Enzymatic Activities of Selected Adaptogenic Plants from South America, Asia, and Africa

**DOI:** 10.3390/molecules28166004

**Published:** 2023-08-10

**Authors:** Jakub Gębalski, Milena Małkowska, Filip Graczyk, Artur Słomka, Elżbieta Piskorska, Dorota Gawenda-Kempczyńska, Aneta Kondrzycka-Dąda, Anna Bogucka-Kocka, Maciej Strzemski, Ireneusz Sowa, Magdalena Wójciak, Sebastian Grzyb, Krystian Krolik, Aneta A. Ptaszyńska, Daniel Załuski

**Affiliations:** 1 Department of Pharmaceutical Botany and Pharmacognosy, Ludwik Rydygier Collegium Medicum, Nicolaus Copernicus University, 85-094 Bydgoszcz, Poland; malkowskamilena7@gmail.com (M.M.); filip.graczyk@cm.umk.pl (F.G.); dgawenda@cm.umk.pl (D.G.-K.);; 2 Department of Pathophysiology, Ludwik Rydygier Collegium Medicum, Nicolaus Copernicus University, 85-094 Bydgoszcz, Poland; artur.slomka@cm.umk.pl; 3 Department of Pathobiochemistry and Clinical Chemistry, Ludwik Rydygier Collegium Medicum, Nicolaus Copernicus University, 85-094 Bydgoszcz, Poland; 4 University of Social Sciences, 11 Łucka Str., 00-842 Warsaw, Poland; 5 Department of Biology and Genetics, Medical University of Lublin, 20-093 Lublin, Poland; 6 Department of Analytical Chemistry, Medical University of Lublin, 4a Chodzki Str., 20-093 Lublin, Poland; 7 College of Engineering and Health in Warsaw, Bitwy Warszawskiej 1920 r. 18 Str., 02-366 Warsaw, Poland; 8 Department of Immunobiology, Institute of Biological Sciences, Faculty of Biology and Biotechnology, Maria Curie-Skłodowska University, Akademicka 19 Str., 20-033 Lublin, Poland

**Keywords:** nutripharmacology, tyrosinase, hyaluronidase, acetylcholinesterase, adaptogens, DPPH, ABTS, ferrozine, ion chelation, polyphenols, flavonoids

## Abstract

Despite the fact that there are many studies related to the adaptogenic and pro-healthy activities of plant-based compounds, there are some adaptogenic plants whose activities are not fully known, especially those coming from the wild regions of Asia, Africa, and South America. The aim of these studies was to examine the contents of non-nutritional compounds, such as polyphenols, flavonoids, and phenolic acids in ten adaptogenic species (*Astragalus membranaceus* (AM), *Uncaria rhynchophylla* (UR), *Polygonum multiflorum* (PM), *Angelica sinensis* (AS), *Andrographis paniculatea* (AP), *Tinospora cordifolia* (TC), *Uncaria tomentosa* (UT), *Pfaffia paniculate* (PP), *Sutherlandia frutescens* (SF), and *Rhaponticum carthamoides* (RC)). Considering biological activity, their antioxidant (DPPH, ABTS, FRAP, and ferrous-ion-chelating ability assays), anti-acetylcholinesterase, anti-hyaluronidase, and anti-tyrosinase activities were evaluated. The richest in polyphenols, flavonoids, and phenolic acids was UR (327.78 mg GAE/g, 230.13 mg QE/g, and 81.03 mg CA/g, respectively). The highest inhibitions of acetylcholinesterase, hyaluronidase, and tyrosinase were observed for TC, UR, and PM, respectively. In the case of antioxidant properties, extract from PM appeared to most strongly reduce DPPH, extract from UR inhibited ABTS, and extract from SF showed the best chelating properties. It should be noted that a particularly interesting plant was *Ulcaria rhynchophylla*. The results mean that there were compounds in UR with broad biological activities, and this species should be explored in more detail. Additionally, our results justify the traditional use of these species in the nutripharmacological or ethnopharmacological care systems of different regions.

## 1. Introduction

Adaptogenic plants belong to a diversified group used to treat many diseases. In 1947, Nikolai Lazarev defined an adaptogen as a remedy that allows an organism to counteract unfavorable physical, chemical, and biological stress effects. In 1968, Dr. Israel I. Brekhman and Dr. I. V. Dardymov defined an adaptogen as a medicine, which should act with no toxicity for humans, should increase resistance to multiple stressors, and should normalize the effects of stressors on physiology. Nevertheless, there is currently no consistent definition of an adaptogen. According to traditional Chinese medicine, adaptogens strengthen and stimulate a body’s immune and defense functions; in Ayurveda, they cause rejuvenation. In Western medicine, they are used to regulate the hypothalamic–pituitary–adrenal axis (HPA) and sympathetic nervous system [1,2].

Additionally, most adaptogens have antioxidant properties. The activity of a plant-based adaptogen is related to its chemical compounds, which are very diversified. It is said that there are a few groups of compounds responsible for the adaptogenic and nutritional values of plant adaptogens, e.g., triterpenoid saponins (*Panax ginseng* ginsenosides; eleutherosides in *Eleutherococcus senticosus*); phytosterols and ecdysones (*Rhaponticum carthamoides*); and lignans (*Schisandra chinensis*). Some adaptogenic plants are used in China as food with great health benefits. In this place, it should be noticed that the roots of *Glycyrrhiza glabra* L. are served in China after frying in bee honey to stimulate the immune system. It is generally known that some compounds, like polyphenols, are suitable for the human body [3,4,5,6].

One of the mechanisms of disease treatment is the inhibition of enzymes. Hyaluronidases (HYALs) belong to the hydrolase responsible for hyaluronic acid (HA) degradation. They are produced by bacteria and sperm, facilitating their entry into egg cells, as well as by neutrophils. They are also present in animal venoms. Overproduction of melanin leads to freckles, age spots, or discoloration after a sunburn. Tyrosinase (TYR) is involved in melanin production, a natural pigment that is synthesized by most organisms. Acetylcholinesterase (AChE) is an enzyme that breaks down one of the primary neurotransmitters, acetylcholine, into choline and acetic acid residue. Inhibition of AChE activity increases cholinergic relay, positively affecting loss symptoms concerning cognitive processes in patients with dementia associated with Alzheimer’s and Parkinson’s [7,8,9].

There are over 8000 polyphenols, and their wide activities have been explored in different models (in vitro and in vivo). Animal, human, and epidemiologic studies have shown that various polyphenols have antioxidant and anti-inflammatory properties that can have preventive and/or therapeutic effects for cardiovascular diseases, neurodegenerative disorders, cancer, and obesity. There are many mechanisms related to the protective effect of polyphenols against free radicals, i.e., by forming stabilized chemical complexes or by producing hydrogen peroxide, which can help to regulate immune response actions [10,11,12]. 

Because polyphenols constitute a significant group of non-nutritional diet constituents, their amounts in foods should be regulated, and recommendations for consumption should be unified. Interest in plant-based adaptogens over the past few years has dramatically increased, and for this reason, it is essential to perform comprehensive research on their compounds and activities using one research model.

We hypothesize that adaptogenic plants may be used not only in ethnomedicine but also as ingredients in food with nutripharmacological activities. For this reason, we choose as model plants ten adaptogenic plants native to three continents, i.e., South America, Asia, and Africa. We explore *Astragalus membranaceus* root (AM), the herb *Uncaria rhynchophylla* (UR), *Polygonum multiflorum* root (PM), *Angelica sinensis* root (AS), the herb *Andrographis paniculate* (AP), *Tinospora cordifolia* (TC), *Uncaria tomentosa* bark (UT), *Pfaffia paniculate* root (PP), *Sutherlandia frutescens* fruit (SF), and *Rhaponticum carthamoides* root (RC). 

To confirm this hypothesis, a wide panel of analyses is conducted, including phytochemical and nutripharmacological ones (antioxidant, anti-tyrosinase, anti-hyaluronidase, and anti-acetylcholinesterase).

## 2. Results and Discussion

### 2.1. Chemical Compositions and Yields of Extraction

Polyphenols (flavonoids, phenolic acids, chalcones, lignans, anthocyanins, and tannins) are natural compounds that are widely spread in the plant world, exhibiting a variety of biological activities. As shown in Table 1, the yields of extraction were different and dependent on the type of raw material. The highest yield of extraction was obtained for AS, with the lowest one for TC. However, there was no correlation between the yield and the polyphenol content or activity. This may mean that there are compounds other than polyphenols whose activities are associated with pharmacological effects. It has been reported in the literature that the extraction of UR hooks resulted in a yield of 6.71%.

Also, the content of polyphenols varied in the range of 80.14 ± 1.2–327.78 ± 26.27 mg GAE/g, the total flavonoid content was in the range of 20.9 ± 0.67–230.13 ± 40.86 mg QE/g, and the total phenolic acid content was in the range of 1.1 ± 0.00–81.0 ± 11.4 mg CAE/g. The highest TPC, TFC, and TPAC values were observed in UR. 

The contents of polyphenols depend on different factors, and in some cases, it is not possible to make comparisons because of the different conditions of analysis and extract preparation. Additionally, the plants tested in this research are not fully explored in terms of polyphenol content. There are reports in the literature on these compounds’ contents in UR and AM, respectively. Different results were obtained by extracting UR hooks with 70% EtOH, followed by fractionation with hexane, ether, dichloromethane (DCM), ethyl acetate (EtOAc), and aqueous solution. The TPC and TFC in the 70% EtOH extract were 241.9 mg GAE/g and 33.5 mg QE/g, respectively. The TPC was the highest in the EtOAC fraction at 373.79 mg GAE/g, whereas the TFC was the highest in the ethanol extract at 33.5 mg QE/g [13]. The differences in the results are due to the different methods of chemical composition determination and extraction used in the studies.

### 2.2. Antioxidant Properties 

#### 2.2.1. Determination of DPPH and ABTS Assays

**Figure 1:**
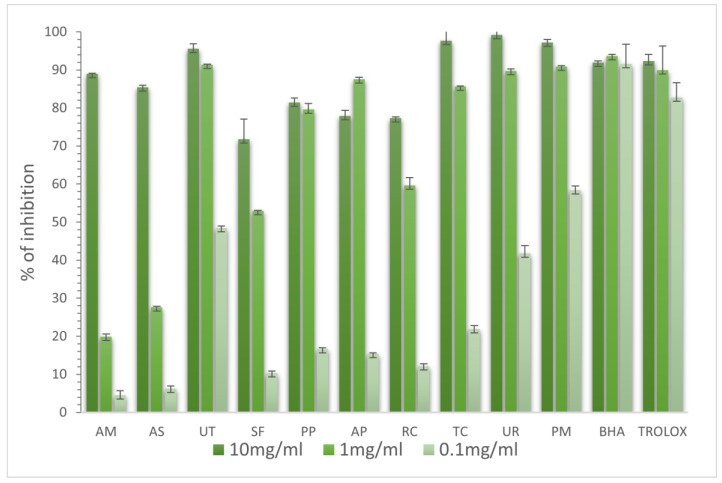
Antioxidant activities of selected adaptogenic plants via ABTS method. The results are presented as the percentage of inhibition of ABTS radicals.

**Figure 2:**
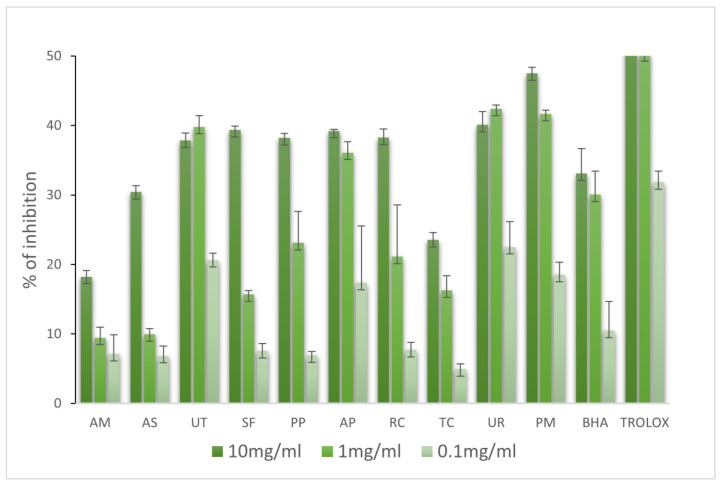
Antioxidant activities of selected adaptogenic plants via DPPH method. The results are presented as the percentage of inhibition of DPPH radicals.

**Figure 3:**
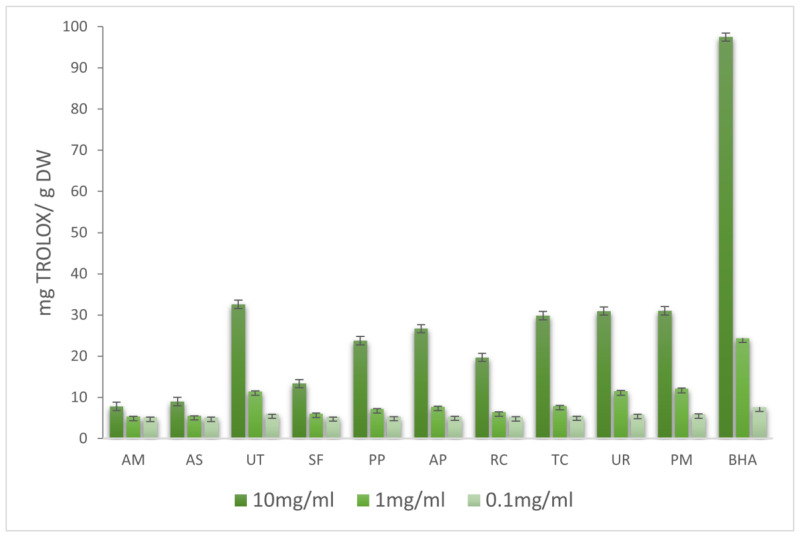
Antioxidant activities of selected adaptogenic plants via FRAP method. The results are expressed as mg Trolox equivalent per gram of sample.

**Figure 4:**
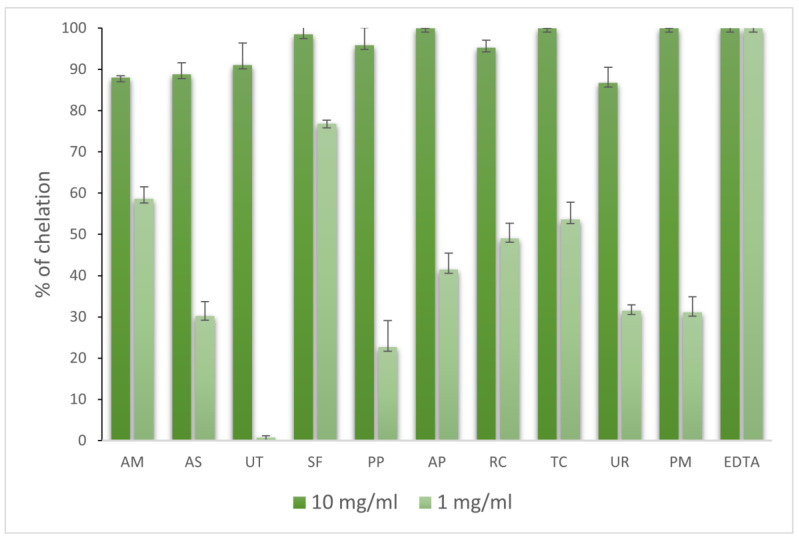
Inhibition of Fe^2+^–ferrozine formation (%).

Several methods allowed us to better define the mechanisms of action of antioxidants in the extracts. To evaluate the antioxidant capacities of the extracts, several in vitro methods were undertaken, namely, DPPH, ABTS, FRAP, and ferrous ion-chelating ability assays. The antioxidant activities of extracts were determined in three concentrations (10, 1, and 0.1 mg/mL). The obtained results are summarized in Figure 1, Figure 2, Figure 3 and Figure 4.

For the ABTS assay, the extracts from UT, AP, UR, and PM at a 1 mg/mL concentration had similar activity to BHA and Trolox. The results are reported in Figure 1. High activities in scavenging DPPH^•^ radicals were shown by the UT, AP, UR, and PM extracts, whose activities were more elevated than BHA in each concentration. Regardless of the concentration used, the activity of Trolox was the highest. The results are reported in Figure 2. There are reports in the literature on thousands of data regarding DPPH reduction; however, these results vary because of the chemical compositions of extracts and the concentrations used. For instance, Jain et al., prepared ethanolic extract from *Tinospora cordifolia* stem (TC) using Soxhlet extraction. The IC_50_ values for ABTS and DPPH were 106.86 and 85.48 μg/mL, respectively [14]. In the case of *Uncaria rhynchophylla* (UR), in a test with ABTS and DPPH the IC_50_ values were equal to 23.52 and 8.70 μg/mL, respectively [15]. Not many adaptogenic plants have been explored in detail including a few organs from one species. Very interesting results were provided by Rafat et al. [16], who analyzed the free-radical-scavenging potential of AP. It appeared that the fruit extract strongly reduced DPPH (88.13%), followed by the leaf extract (86.87%), and the stem extract (80.48%).

#### 2.2.2. Ferric-Reducing Antioxidant Power (FRAP) Assay

As assessed with the FRAP method, the reducing potential varied largely among the investigated extracts. The results are presented in Figure 3. UT showed the most potent reducing properties at 10 mg/mL (32.58 mg Trolox/g DW). The values of TC (29.86 mg Trolox/g DW), UR (30.95 mg Trolox/g DW), and PM (31.01 mg Trolox/g DW) were similar to that of UT. PP, AP, and RC reduced iron ions at 23.76, 26.67, and 19.67 mg Trolox/g DW, respectively. At a 1 mg/mL, UT, UR, and PM showed the highest reducing capacities (11.46, 11.49, and 12.09 mg Trolox/g DW, respectively). At a concentration of 0.1 mg/mL, the range of reducing power was from 5.14 to 5.92 mg Trolox/g DW. Ethanolic extract prepared from UT leaves showed moderate antioxidant activity (2.9 mM FeSO_4_/mg) [17]. The extract made from TC stem showed strong protective properties against iron (III) ions (197.5 FSE/g DW) [18]. The results show that water extracts (2.039 ± 0.06 mM FeSO_4_ eq./mg extracts) and ethanol extracts (2.417 ± 0.11 mM FeSO_4_ eq./mg extracts) both had higher FRAP antioxidant activities than those of BHT (1.284 ± 0.16 mM FeSO_4_ eq./mg extracts) [19]. The water extract of the stem of PM demonstrated intense antioxidant activity (498 μM Fe (II)/g), as well as the stem of PM (343 μM Fe (II)/g) [20].

#### 2.2.3. Ion Chelation Assay

Compounds present in the extracts showed high chelating abilities at a concentration of 10 mg/mL (Figure 4). SF, PP, AP, RC, TC, and PM demonstrated substantial chelating properties. At a 1 mg/mL concentration, chelating activity was most significant for SF, with weaker properties shown by AM, RC, and TC. No chelation was observed for UT (at 1 mg/mL). The strong chelation activity of SF is confirmed by Tobwala with an extract obtained with the hot water chelating of iron (II) ions at 40% (for EDTA, about 50%) [21].

### 2.3. Enzymatic Inhibition

Plants have been used as a source of medicine for centuries. The presence of a wide variety of chemical structures allows for the discovery of many drugs. Important plant-derived compounds include morphine, galantamine, atropine, vincristine, vinblastine, paclitaxel, and ephedrine. Adaptogenic plants, due to their diverse compositions, are a great source of new molecules, which may stimulate the activities of different enzymes. This is especially desirable in the prevention or treatment of diseases that result from the overactivity of some enzymes, i.e., acetylcholinesterase, tyrosinase, or hyaluronidase. 

Alkaloid compounds are probably responsible for the activity of extracts against acetylcholinesterase. The structures of these compounds allow interaction with the active center. Flavonoids containing a γ-pyrronium grouping may be responsible for inhibiting tyrosinase. Activity against hyaluronidase is shown by many plant compounds, such as alkaloids, flavonoids, phenolic acids, saponins, and tannins.

#### 2.3.1. Acetylcholinesterase Inhibition Assay

**Figure 5:**
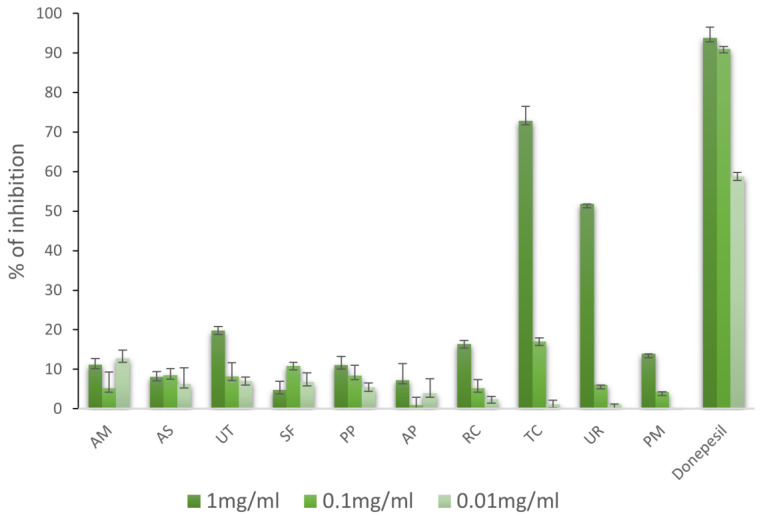
Activities of extracts against acetylcholinesterase (%).

The activities of methanolic extracts against AChE are shown in Figure 5. As shown, all the extracts were rather weak as AChE inhibitors, and high doses were needed. The most potent inhibitory activities at a concentration of 10 mg/mL were shown by TC and UR (72.80 and 51.80%, respectively). The other extracts showed weak AChE inhibition. None of the extracts showed more potent activity against AChE than donepezil. There were not many results regarding these species and their anti-AChE activities. The methanolic extract of UR leaves (1 mg/mL) inhibited the enzyme by 90%, which probably resulted from the presence of geissoschizine methyl ether (IC_50_ = 3.7 μg/mL). However, this compound inhibited the enzyme more weakly than physostigmine (IC_50_ = 0.013 μg/mL). Six alkaloids (vallesiachotamine, histamine, hirsute, isorhynchophylline, cisocorynoxeine, and corynoxeine) are responsible for this effect to a lesser extent [22,23]. Another report provided by Chowdhury et al., claimed that an aqueous extract of *U. tomentosa* bark showed a maximum AChE inhibition of 76.2% at 0.4 mg/mL of final concentration with an IC_50_ = 0.112 mg/mL [24]. In the research of Jiang et al., it appeared that geissoschizine methyl ether inhibited AChE at the level of 50% in a dose of 23.4 μM [25]. In turn, an extract made from the stem of TC inhibited AChE with an IC_50_ value of 38.36 μg/mL. Columbine, one of the active compounds of TC, exhibited a weaker inhibition for the IC_50_ value of 1.29 mg/mL in a comparison to the eserine (IC_50_ = 0.5318 mg/mL) used as a standard. In another study, the tinosporidium (IC_50_ = 13.45 μg/mL) and 8-hydroxytinosporidium (IC_50_ = 46.71 μg/mL) obtained from a methanolic extract were also identified as AChE inhibitors. In turn, oxoglaucine, lyriodenine, and N-formylanonine showed weak anti-AChE activity, with IC_50_ values of 0.8033, 0.8076, and 0.8190 mg/mL, respectively [26,27]. 

Recently, a new compound was isolated from the stem of TC, and the molecule was identified as rel (2S,3S,4R,16E)-2-[(2′R)-2′-hydroxynonadecanoylamino]-heneicosadec-16-ene-1,3,4-triol and demonstrated quite good AChE inhibitory activity (IC_50_ = 0.055 mg/mL) when compared to eserine (IC_50_ = 0.009 mg/mL) [28].

Santoro et al., reported the anti-AChE activity of an AM root ethanolic extract with an IC_50_ = 11.7 μg/mL [29]. Despite the weak activity of the AM extract, there were some reports about isolated compounds from it claiming anti-AChE potential: calycosin-7-*O*-β-D-glucoside (44.22 μg/mL), pratensein7-*O*-β-d-glucoside (IC_50_ = 48.09 μg/mL), formononetin-7-*O*-β-d-glucoside (IC_50_ = 49.69 μg/mL), calycosin (IC_50_ = 46.96 μg/mL), genistein (IC_50_ = 45.13 μg/mL), and formononetin (IC_50_ = 44.83 μg/mL) [30]. Stępnik et al., reported the AChE inhibitory potential of astragalosides found in the roots of AM, with the IC_50_ values for astragalosides II, III, and IV (5.9, 4.2, and 4.0 μM, respectively) [31].

Regarding AP extracts, Mukherjee et al., also reported a weak inhibition of acetylcholinesterase with an IC_50_ value of 222.41 ± 19.87 µg/mL for a hydroalcoholic extract [32].

#### 2.3.2. Tyrosinase Inhibition Assay

**Figure 6:**
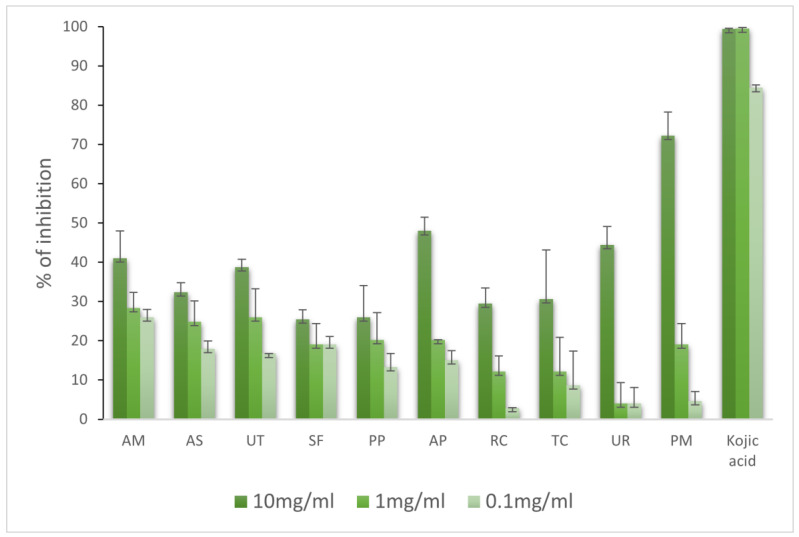
Activities of extracts against tyrosinase (%).

The assay was carried out at three different extract concentrations: 10, 1, and 0.1 mg/mL. The results showed a dose-dependent inhibition, with the highest inhibition for 10 mg/mL (Figure 6). The most active extract was PM (72.25%), followed by AP and UR (above 40%).

Several studies have demonstrated the multidirectional whitening effects of PM extracts. Inhibitory activity against tyrosinase was shown by (E)-2,3,5,4′-tetrahydroxystilbene-2-*O*-β-d-glucoside (THSG) isolated from PM. THSG inhibited tyrosinase in a dose-dependent manner, with an IC_50_ value of 100 μg/mL. In addition, THSG also strongly inhibited melanin production in B16 melanoma cells induced by forskolin, which indicated the potential stimulation of melanogenesis [33]. Based on the results obtained by Hamid et al. [34], it may be stated that AP extract inhibited tyrosinase more strongly than an isolated compound, andrographolide (IC_50_ = 0.749 and 2.441 μg/mL, respectively). Kojic acid (IC_50_ = 19.985 μg/mL) was used as the standard. Also, AM, due to the presence of flavonoids (formononetin) and saponins, has multidirectional effects on the production of melanin. Lee et al., isolated calycosin-7-*O*-β-d-glucopyranoside, which was responsible for anti-tyrosinase activity (IC_50_ = 68.1 μM). As standards, kojic acid and arbutin were used (IC_50_ = 79.5 and 125.1 μM, respectively) [35,36]. Dong et al. [37] determined the effect of a 70% ethanolic extract of UR on tyrosinase activity and melanin synthesis. The extract of 1 mg/mL (60% activity of tyrosinase) inhibited tyrosinase more strongly than kojic acid (65% activity of tyrosinase), as well as significantly decreasing the cellular melanin content. In this place, it should be mentioned that inhibition is strongly dependent on the chemical groups bound to a ring. Despite many results related to this subject, in the majority it is impossible to make comparisons because of a lack of information about the enzyme unit activity or insufficient chemical analysis.

#### 2.3.3. Hyaluronidase Inhibition Assay

**Figure 7:**
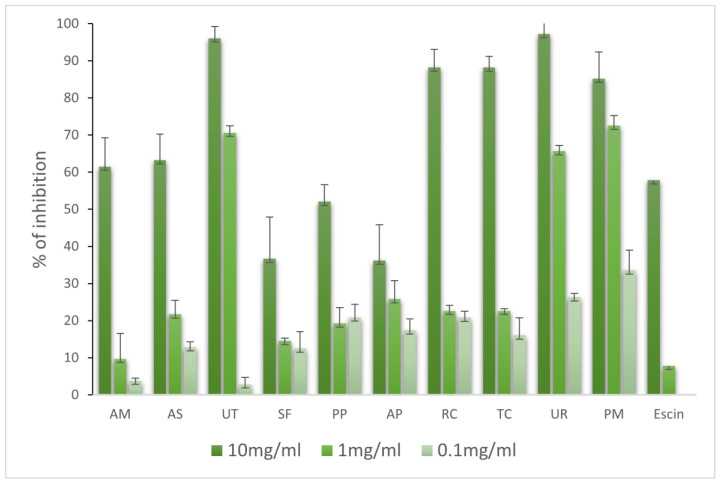
Activities of extracts against hyaluronidase (%).

The activities of 10 adaptogenic extracts against hyaluronidase were determined at concentrations of 10, 1.0, and 0.1 mg/mL. As reported in Figure 7, all the extracts contained phytochemicals with moderate activity, and high doses were needed to obtain significant inhibition. Under consideration should be the extracts from PM (72.52%), UT (70.60%), and UR (65.66%), which at 1 mg/mL, showed the most potent activities when compared to the dose. Of course, the extracts from UR (97.23%) and UT (96.06%) were found to have the strongest inhibition of hyaluronidase; however, they needed to use a high concentration of 10 mg/mL. Our study showed for the first time the strong inhibitory activities of *Uncaria tomentosa*, *Uncaria rhynchophylla,* and *Polygonum multiflorum* against bovine hyaluronidase. 

There are not many data regarding these species and their anti-HYAL activity. However, Kang et al., examined various plant species, including AM and PM, reporting that only AM ethanolic root extract inhibited hyaluronidase at 76.2% [38]. In another study, AM and PM water extracts and ethanol extracts inhibited hyaluronidase (7900 U/mL) at 11.6% and 57.5% for AM and 18.7% and 50.4% for PM, respectively. The AM extract may not have a high inhibiting potential of hyaluronidase; however, there are reports that have indicated that AM leaf extracts may increase the content of hyaluronic acid in tissues, which can also prevent spreading infections and inflammatory processes [39,40,41].

Our results show that the *Andrographis paniculatae* extract did not exhibit a higher inhibitory potential amongst other species. However, some results indicate that AP methanolic extracts (100–400 µg/mL) inhibited hyaluronidase to the extent of 17–52% [42]. Sivakumar et al., proved that methanol leaf AP extract at a concentration of 250 μg/mL inhibited hyaluronidase at 32 ± 6% [43].

### 2.4. Statistical Analysis and Correlation

The contents of TPC and TPAC showed statistically significant correlations with most of the applied antioxidant tests for the majority of the extract concentrations used (Table 2). The only exceptions were for the DPPH test with a 10 mg/mL extract concentration and for the CA test with a 1 mg/mL extract concentration, where there were no statistically significant correlations with any of the studied active compounds. In the case of antioxidant activity, the TFC content correlated with the results of the FRAP assay for all the extract concentrations, as well as with the ABTS and CA assays with the 10 mg/mL extract concentrations. For enzymatic inhibition, statistically significant correlations with TPC, TFC, and TPAC were obtained only for the AChE test at 1 mg/mL, with TPC and TFC for the HYAL test at 0.1 mg/mL, and with TFC for the TYR test at 1 mg/mL.

**Figure 8:**
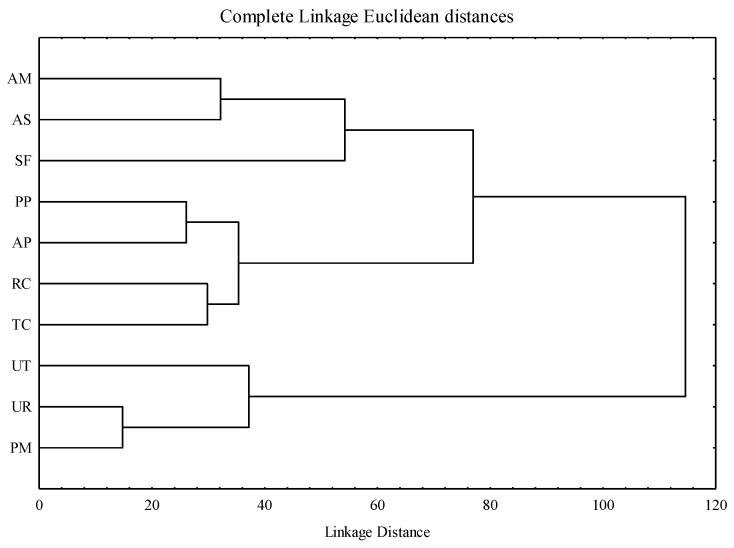
Hierarchical cluster analysis of adaptogenic plant samples based on antioxidant activity and enzymatic inhibition.

A hierarchical cluster analysis (Figure 8) was conducted based on the antioxidant activities and enzymatic inhibitions of the adaptogenic species. The results suggested the presence of three distinct groups among the analyzed taxa. Group I comprised three species: AM, AS, and SF; group II consisted of four species: PP, AP, RC, and TC; and group III included three species: UT, UR, and PM.

**Figure 9:**
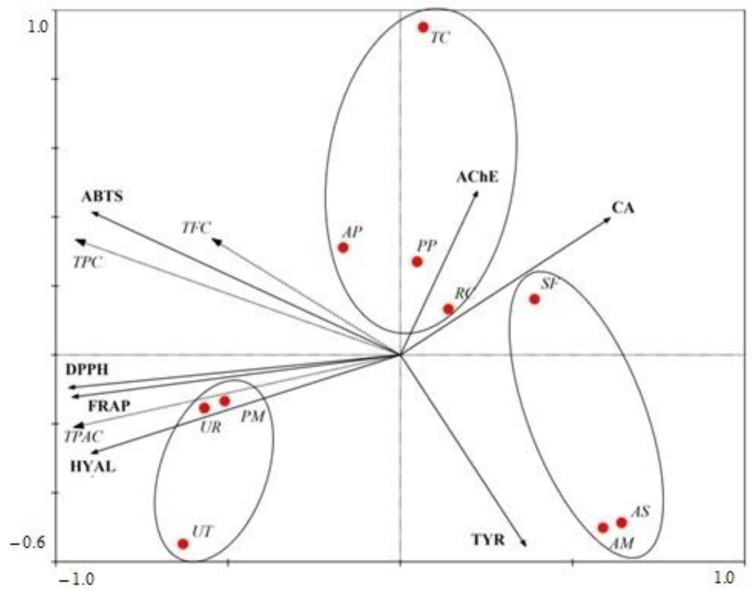
Redundancy analysis (RDA) of the samples of adaptogenic plants based on content of chemical composition, antioxidant activity, and enzymatic inhibition. Eigenvalues: *x*-axis—0.696, *y*-axis—0.131, *p*—0.002 (Monte Carlo test).

The same groups were also identified in the redundancy analysis (RDA) based on the contents of active compounds and the investigated activities: antioxidant activity and enzymatic inhibition (Figure 9). The eigenvalues for the first and second canonical axes were 0.696 and 0.131, respectively. A Monte Carlo permutation test showed that the content of the chemical composition was responsible for the variability in the antioxidant and anti-enzymatic activities of the studied adaptogenic species, with a statistical significance of *p* = 0.002 for the first axis and *p* = 0.002 for all the axes. The distribution of species along the *x*-axis was determined by the contents of TPAC (correlation with *x*-axis: −0.9278) and TPC (correlation with *x*-axis: −0.9224).

In the ordination space, species from group I were located between the vectors represented by CA and TYR, while species from group II were clustered near the HYAL vector. The third group of species was associated with higher content of TPAC and stronger inhibition of AChE, as well as increased antioxidant activity against DPPH and FRAP.

## 3. Materials and Methods

### 3.1. Chemicals and Reagents

1,3,5-Tri(2-pyridyl)-2,4,6-triazine (TPTZ), 2,2-diphenyl-1-picrylhydrazyl (DPPH), 2,2′-azinobis-(3-ethylbenzthiazoline-6-sulfonic acid) (ABTS), 3-(2-Pyridyl)-5,6-diphenyl-1,2,4-triazine-p,p′-disulfonic acid monosodium salt hydrate (ferrozine), Folin−Ciocalteu reagent ascorbic acid, 6-hydroxy-2,5,7,8-tetramethylchroman-2-carboxylic acid (Trolox), 2(3)-t-Butyl-4-hydroxyanisole, 2(3)-t-Butylhydroquinone monomethyl ether (BHA), escin, donepezil, koji acid, iron(II) chloride tetrahydrate (FeCl_2_ × 4H_2_O, iron(III) chloride (FeCl_3_), potassium persulfate, buffers for enzymatic analysis, L-tyrosine, L-DOPA, tyrosine from mushrooms, acetylcholine (ACh), acetylcholinesterase, 5,5′-Dithiobis(2-nitrobenzoic acid) (DTNB), hyaluronidase from bovine testes, hyaluronic acid (IV), and hexadecyltrimethylammonium bromide (CTAB) were purchased from Sigma-Aldrich Corp (Saint Louis, MO, USA). Solvents used for extraction were purchased from Avantor Performance Materials, Poland S.A. (Gliwice, Poland).

### 3.2. Plant Material

Adaptogenic plants from South America, Asia, and Africa were studied. The plant materials were bought at a shop (MagicznyOgród, Muszyna, Poland). Each of the raw materials was certified for quality. The plants came from the following countries: China—*Astragalus membranaceus* Bunge root, *Polygonum multiflorum* (Thunb.) Moldenke root, *Angelica sinensis* (Oliv.) Diels root, and *Uncaria rhynchophylla* (Miq.) Jacks. Herb; India—*Andrographis paniculate* (Burm.f.) Nees herb and *Tinospora cordifolia* (Willd.) Hook. f. and Thomson herb; Peru—*Uncaria tomentosa* (Willd. ex Schult.) DC. bark; Brazil—*Pfaffia paniculate* (Mart.) Kuntze root; South Africa—*Sutherlandia frutescens* (L.) R.Br. fruit; and Russia—*Rhaponticum carthamoides* (Willd.) Iljin root.

### 3.3. Extraction

An amount of 10 g of raw material was extracted with 50 mL of 75% MeOH and sonicated for 30 min. The extraction was repeated thrice, and the filtrate was concentrated with a vacuum evaporator at 30 °C. Next, the extracts were lyophilized and stored in a fridge at −20 °C. The extraction yield was calculated based on the dry weight of the extract (%).

### 3.4. Chemical Composition

#### 3.4.1. Determination of Total Phenolic Content (TPC)

Total phenolic content was determined using the Folin–Ciocalteu method with slight modification [44]. Briefly, 25 μL of extract (1 mg/mL in MeOH) was mixed with 25 μL of Folin−Ciocalteu reagent (diluted in pure water, 1:3). Next, 200 μL of distilled water was added, and the mixture was then incubated for 5 min. After that, 25 μL of sodium carbonate (20%) solution was added and incubated in the dark at room temperature for an hour. The absorbance was measured at 750 nm. The results for TPC are expressed in milligrams of gallic acid (GA) equivalent (GAE) per gram of the sample (mg GAE/g sample).

#### 3.4.2. Determination of Total Flavonoid Content (TFC)

Total flavonoid content was determined using a method based on the reaction between AlCl_3_ and flavonoids [45]. Briefly, 25 μL of extract (1 mg/mL in MeOH) was mixed with 75 μL of EtOH. After that, 10 μL each of aluminum chloride (10%) and potassium acetate (1 M) were added. An amount of 130 μL of distilled water was added. Next, the mixture was incubated for 30 min. The absorbance was measured at 510 nm. The results for TFC are expressed in milligrams of quercetin (Q) equivalent (QE) per gram of the sample (mg QE/g sample).

#### 3.4.3. Determination of Total Phenolic Acid Content (TPAC)

Total phenolic acid content was determined according to the method described in Polish Pharmacopeia VI [46]. In short, 25 μL of extract (1 mg/mL in MeOH) was mixed with 150 μL of distilled water. Next, 25 μL of HCl (0.5 M) and 25 μL of Arnov’s reagent (10.0 g of sodium molybdate and 10.0 g of sodium nitrite in 100 mL distilled water) were added. After that, 25 μL of NaOH (1 M) was mixed, and the mixture was immediately measured at 492 nm. The results for TPC are expressed in milligrams of caffeic acid (CA) equivalent (CAE) per gram of the sample (mg CAE/g sample).

### 3.5. Antioxidant Properties 

#### 3.5.1. ABTS Free-Radical-Scavenging Activity

ABTS free radical scavenging was conducted following the method of Wu et al. [47]. Briefly, working ABTS^+^ solution was prepared by mixing 10 mL of ABTS (7 mM in H_2_O) and 10 mL of potassium persulfate (2.45 mM in H_2_O), which was further incubated in the dark for 12 h. Next, the ABTS+ solution was diluted with water to obtain an absorbance of 0.700 ± 0.03 at 405 nm. After that, 10 μL of extract (1 mg/mL/0.1 mg/mL/0.01 mg/mL) was mixed with 190 μL of ABTS+ solution and incubated for 30 min. After incubation, the absorbance was measured at 405 nm. As controls, Trolox and BHA were used. The antioxidant activity was calculated using the following equation:

$${\%}_{INH}=\left(\frac{A_{S}-A_{C}}{A_{C}}\right)*100\%$$

A_S_—the absorbance for sample + ABTS;

A_C_—the absorbance without sample + ABTS.

#### 3.5.2. DPPH Free-Radical-Scavenging Activity

DPPH free-radical-scavenging was conducted following the method of Naseer et al. [48]. In short, working DPPH^+^ solution was prepared by dissolving 24 mg of DPPH in 100 mL of distilled water. Next, the DPPH^+^ solution was diluted with methanol to obtain an absorbance of 0.900 ± 0.03 at 515 nm. After that, 10 μL of extract (1 mg/mL/0.1 mg/mL/0.01 mg/mL) was mixed with 190 μL of DPPH^+^ solution and incubated for 60 min. After incubation, the absorbance was measured at 515 nm. As controls, Trolox and BHA were used. The antioxidant activity was calculated using the following equation:

$${\%}_{INH}=\left(\frac{A_{S}-A_{C}}{A_{C}}\right)*100\%$$

A_S_—the absorbance for sample + DPPH;

A_C_—the absorbance without sample + DPPH.

#### 3.5.3. Ferric-Ion-Reducing Antioxidant Power (FRAP) Assay

FRAP assays of different adaptogenic plant extracts were conducted following the method of Sharifi-Rad et al. [49]. Briefly, 10 μL of extract (final concentrations in well: 0.34, 0.034, and 0.0034 mg/mL) was mixed with 290 μL of a working solution consisting of 15 mL of acetate buffer, 1.5 mL of TPTZ solution, and 1.5 mL of FeCl_3_ × 4H_2_O. The mixture was incubated for 30 min. After that, the absorbance was measured at 593 nm. As controls, Trolox and BHA were used. The results for FRAP are expressed in milligrams of Trolox per gram of the sample (mg Trolox/g sample).

#### 3.5.4. Iron (II) Ion Chelation Assay

Ion chelation assays were conducted using the method described by Li et al. [50]. Briefly, 100 µL of extract (final concentrations in well: 3.84 and 0.384 mg/mL) and 150 µL of MeOH were mixed with 5 µL of FeCl_2_ (2 mM). In the next step, 5 µL of ferrozine (5 mM) was added. After incubation, the absorbance was measured at 510 nm. As a positive control, ethylenediaminetetraacetic acid (EDTA) was used. The chelation was calculated using the following equation:

$${\%}_{chel.}=\left(1-\frac{A_{S}}{A_{C}}\right)*100\%$$

A_S_—the absorbance for sample + ferrozine + FeCl_2_;

A_C_—the absorbance without sample + ferrozine + FeCl_2_.

### 3.6. Anti-Enzymatic Panel 

#### 3.6.1. Hyaluronidase Inhibition Assay

Hyaluronidase inhibitor assays were performed in 96-well plates according to a modified method described by Di Ferrante [51] and Studzińska-Sroka [52]. The activities of the compounds/extracts were determined via the precipitation of undigested hyaluronic acid with cetyltrimethylammonium bromide (CTAB). Amounts of 10 μL of sample (final concentrations in well: 0.34, 0.034, and 0.0034 mg/mL), 15 μL of acetate buffer (pH = 5.35), 25 μL of incubation buffer (pH = 5.35; 0.1 mg/mL BSA and 4.5 mg/mL NaCl), and 25 μL of enzyme (30 U/mL, incubation buffer) were mixed. After 10 min of incubation at 37 °C, 25 μL (0.3 mg/mL in acetate buffer; pH = 5.35) of hyaluronic acid solution was added. Afterward, plates were incubated for 45 min at 37 °C. After incubation, undigested HA was precipitated by adding 200 μL of 2.5% CTAB. The plates were kept at 25 °C for 10 min. The intensity of complex formation was measured at 600 nm. To determine the presence of inhibition, the absorbance of solutions without an inhibitor (A_C_) or enzyme (A_T_) was measured. All samples were tested in triplicate. Escin was used as a standard. The hyaluronidase inhibition was calculated using the following equation:

$${\%}_{INH}=\left(\frac{A_{S}-A_{C}}{A_{T}-A_{C}}\right)*100\%$$

A_S_—absorbance of HA + sample + enzyme;

A_C_—absorbance of HA + enzyme;

A_T_—absorbance of HA + sample. 

#### 3.6.2. Tyrosinase Inhibition Assay

Tyrosinase inhibitor assays were performed in 96-well plates according to a modified method described by Sigma-Aldrich [53]. Tyrosinase is the enzyme responsible for converting L-tyrosinase to L-DOPA and L-DOPA to DOPA-quinone, accompanied by the browning of the solution. Briefly, 10 μL of sample (final concentrations in well: 0.5, 0.05, and 0.005 mg/mL), 140 μL of phosphoric buffer (pH = 6.8), and 25 μL of enzyme (125 U/mL in phosphoric buffer; pH = 6.8) were mixed and incubated for 10 min at room temperature. In addition, a control without inhibitor was prepared (Ac). After incubation, to each well 25 μL of L-tyrosine (0.3 mg/mL) was added, and the absorbance was measured at 510 nm (kinetic model, every 5 min). Next, two time points (t_1_ and t_2_) were selected in the linear range of the graph. All samples were tested in triplicate. Kojic acid was used as a standard. The tyrosinase inhibition was calculated using the following equation:

$${\%}_{INH}=\left(\frac{{\Delta A}_{S}-{\Delta A}_{S}}{{\Delta A}_{C}}\right)*100\%$$

A_S_—the difference in absorbance between times t_2_ and t_1_ for sample;

A_C_—the difference in absorbance between times t_2_ and t_1_ for positive control.

#### 3.6.3. Acetylcholinesterase Inhibition Assay

Acetylcholinesterase inhibitor assays were performed in 96-well plates according to the Ellman method [54]. Amounts of 45 μL of enzyme (0.4 U/mL, phosphoric buffer; pH = 7.5) and 5 μL of sample (final concentrations in well: 0.25, 0.025, and 0.0025 mg/mL) were mixed and incubated for 15 min at room temperature. After incubation, 150 μL of solution (154 μL of buffer, 1 μL of substrate, and 0.5 μL of DNTB) was added, and absorbance was measured at two points, t_0_ and t_10_, at the wavelength of 405 nm. All samples were tested in triplicate. Donepezil was used as a standard. The positive control (A_C_) was without an inhibitor. The acetylcholinesterase inhibition was calculated using the following equation:

$${\%}_{INH}=\left(1-\frac{{\Delta A}_{S}}{{\Delta A}_{C}}\right)*100\%$$

A_S_—absorbance of acetylcholine + enzyme + sample and the difference in absorbance between times t_2_ and t_1_ for sample;

A_C_—absorbance of acetylcholine + enzyme and the difference in absorbance between times t_2_ and t_1_ for positive control. 

### 3.7. Statistical Analysis

The results are presented as means and standard deviations (SDs). One-way analysis of variance (ANOVA) and Dunnett’s test were used to assess the statistical significance of the means, with *p* < 0.05 considered significant. The statistical analysis was conducted using the Statistica 12 software package. Given the nature of the data, nonparametric tests were employed to perform the statistical analysis. For the active compounds, the content of a nonparametric analysis of variance (Kruskal–Wallis test) was performed. To assess the strength and direction of associations among active compounds, antioxidant properties, and enzymatic inhibitions, correlation analyses were carried out utilizing Spearman’s rank correlation coefficient. To elucidate the relationships among the active compounds, antioxidant properties, and enzymatic inhibitions of the 10 adaptogenic species, a hierarchical cluster analysis and a redundancy analysis (RDA) were performed. The redundancy analysis (RDA) was performed using CANOCO5 software.

## 4. Conclusions

Adaptogenic plants, due to their well-known safety profiles and diverse chemical compositions, are a valuable source of new pharmacophores in drug design. *Uncaria tomentosa* represented high activity in all the tests, which to the best of our knowledge, was determined for the first time. This species should be taken into consideration in further research regarding active compound isolation. 

## Figures and Tables

**Table 1:** Contents of phenolic compounds (1 mg/mL) and yields of extraction (%). Values are expressed as mean ± SD (*n* = 3).

	TPC (mg GAE/g)		TFC (mg QE/g)		TPAC (mg CAE/g)		Yield of Extraction	
	Mean	SD	Mean	SD	Mean	SD	Mass (g)	(%)
*Astragalus membranacus* (AM)	87.90	6.47	20.90	0.67	1.11	0.00	2.50	25.0
*Angelica sinensis* (AS)	80.14	1.16	55.17	8.81	2.90	0.12	5.29	52.9
*Uncaria tomentosa* (UT)	308.87	9.84	74.44	1.55	70.35	7.87	1.10	11.0
*Sutherlandia frutescens* (SF)	168.23	7.11	79.37	20.44	5.76	0.29	3.30	33.0
*Pfaffia paniculata* (PP)	235.82	8.80	32.77	8.56	14.07	0.97	1.33	13.3
*Andrographis paniculata* (AP)	279.27	4.20	83.85	12.61	31.10	0.71	1.68	16.8
*Rhaponticum carthamoides* (RC)	210.92	8.15	43.53	4.95	13.84	1.65	1.24	12.4
*Tinospora cordifolia* (TC)	280.82	8.30	178.38	35.54	19.81	2.82	0.94	9.4
*Uncaria rhynchophylla* (UR)	327.78	26.27	230.13	40.86	81.03	11.41	1.21	12.1
*Polygonum multiflorum* (PM)	314.60	12.06	114.99	12.80	61.61	2.44	3.00	30.0

TPC = total phenol content, TFC = total flavonoid content, TPAC = total phenolic acid content. Results are expressed as mean ± SD.

**Table 2:** Spearman correlations among chemical compositions, antioxidant activities, and enzymatic inhibitions of the adaptogenic plants. Red values indicate statistical significance at *p* < 0.05.

Activity		Extract Concentration	TPC	TFC	TPAC
Antioxidant activity	ABTS	10	−0.66	−0.81	−0.61
		1	0.93	0.62	0.96
		0.1	0.94	0.62	0.92
	DPPH	10	0.53	0.53	0.54
		1	0.92	0.59	0.95
		0.1	0.66	0.37	0.72
	FRAP	10	0.94	0.65	0.95
		1	0.98	0.75	0.95
		0.1	0.95	0.68	0.94
	CA	10	0.71	0.86	0.65
		1	−0.33	0.05	−0.47
Enzymatic inhibition	AChE	10	0.56	0.31	0.54
		1	0.87	0.70	0.88
		0.1	0.53	0.44	0.47
	HYAL	10	0.62	0.38	0.53
		1	−0.25	0.04	−0.26
		0.1	−0.64	−0.64	−0.54
	TYR	10	0.02	−0.09	0.01
		1	−0.48	−0.66	−0.40
		0.1	−0.61	−0.42	−0.55

## Data Availability

The data presented in this study are available on request from the corresponding author.
